# Further characterization of the zebrafish model of acrylamide acute neurotoxicity: gait abnormalities and oxidative stress

**DOI:** 10.1038/s41598-019-43647-z

**Published:** 2019-05-08

**Authors:** Melissa Faria, Arnau Valls, Eva Prats, Juliette Bedrossiantz, Manuel Orozco, Josep M. Porta, Leobardo Manuel Gómez-Oliván, Demetrio Raldúa

**Affiliations:** 1 Institute for Environmental Assessment and Water Research (IDAEA-CSIC), Jordi Girona, 18, 08034 Barcelona, Spain; 2 Institut de Robòtica i Informàtica Industrial, CSIC-UPC, Barcelona, Spain; 3 Research and Development Center (CID-CSIC), Jordi Girona 18, 08034 Barcelona, Spain; 4 Laboratorio de Toxicología Ambiental, Facultad de Química, Universidad Autónoma del Estado de México, Paseo Colón intersección Paseo Tollocan s/n. Col. Residencial Colón, 50120 Toluca, Estado de México Mexico

**Keywords:** Experimental models of disease, Diseases of the nervous system

## Abstract

Occupational, accidental, or suicidal exposure to acrylamide (ACR) may result in a neurotoxic syndrome. Development of animal models of acrylamide neurotoxicity is necessary for increasing our mechanistic understanding of this syndrome and developing more effective therapies. A new model for acute ACR neurotoxicity has been recently developed in adult zebrafish. Whereas the results of the initial characterization were really promising, a further characterization is needed for testing the construct validity of the model. In this study, the presence of gait abnormalities has been investigated by using *ZebraGait*, software specifically designed to analyze the kinematics of fish swimming in a water tunnel. The results of the kinematic analyses demonstrated that the model exhibits mild-to-moderate gait abnormalities. Moreover, the model exhibited negative scototaxis, a result confirming a phenotype of anxiety comorbid with depression phenotype. Interestingly, depletion of the reduced glutathione levels was found in the brain without a concomitant increase in oxidative stress. Finally, hypolocomotion and positive geotaxis exhibited by this model were fully recovered 5 days after transferring the fish to clean fish-water. All this data support the validity of the ACR acute neurotoxicity model developed in adult zebrafish.

## Introduction

Acrylamide (ACR) is a chemical widely used in the industry, the wastewater treatment processes, and the chemical grouting^1,2^. Occupational, accidental, or suicidal exposure of human to ACR results in a neurotoxic syndrome characterized by gait abnormalities, skeletal muscles weakness, numbness of the extremities, and other symptoms related to polyneuropathy^3–5^. Whereas the mechanism of action behind ACR neurotoxicity is still unclear, the most accepted hypothesis is that the primary site of action is at the presynaptic part of the nerve terminals, and the molecular initiating event of this process (MIE) is the formation of adducts with specific sulfhydryl thiolate sites located on specific proteins directly involved in the synaptic vesicles recycling^6^. As the cysteine thiolate groups preferentially targeted by ACR are often located within the active site of the synaptic proteins, adduction of these sites impairs the synaptic function^6^. Reduced glutathione (GSH) depletion and concomitant onset of oxidative stress in the brain have also been suggested as a potential mechanism for ACR neurotoxicity^7–10^. Thus, there is a need of validated animal models of acrylamide neurotoxicity for increasing our current understanding of the mechanism of action and developing effective therapies for the clinical management of the patients.

Zebrafish (*Danio rerio*) is a vertebrate model increasingly used in biomedical research, including the development of human neurotoxicology models, as this species exhibits a similar overall nervous system organization to humans and similar neurotransmitter systems^11–13^. Recently, we developed an ACR acute neurotoxicity model in adult zebrafish by exposing animals to 0.75 mM ACR in water for 3 days^14^. Although the initial characterization at transcriptional and proteome level suggested the impairment of the presynaptic vesicle cycling function, the potential involvement of oxidative stress was not explored. Moreover, the presence of gait abnormalities, one of the main neurological signs of ACR neurotoxicity in humans, was not determined in the developed model. Thus, a further characterization of the zebrafish model of ACR neurotoxicity is needed as a part of its construct validity.

In this study we present *ZebraGait*, a new kinematic analysis software able to provide quantitative information of the motor function during the fish swimming in a water tunnel. By using *ZebraGait*, the presence of gait abnormalities in the developed zebrafish model has been determined. Moreover, the dark-light test (DLT) paradigm has been used for a further analysis of the behavioral phenotype. Finally, the severity of the phenotype has been evaluated by determining both the presence of oxidative stress in the brain and the reversibility of the behavioral phenotype. The different results presented in this manuscript support the validity of the adult zebrafish model of acrylamide neurotoxicity.

## Results and Discussion

### Zebrafish model of ACR poisoning exhibits gait abnormalities

**Figure 1:**
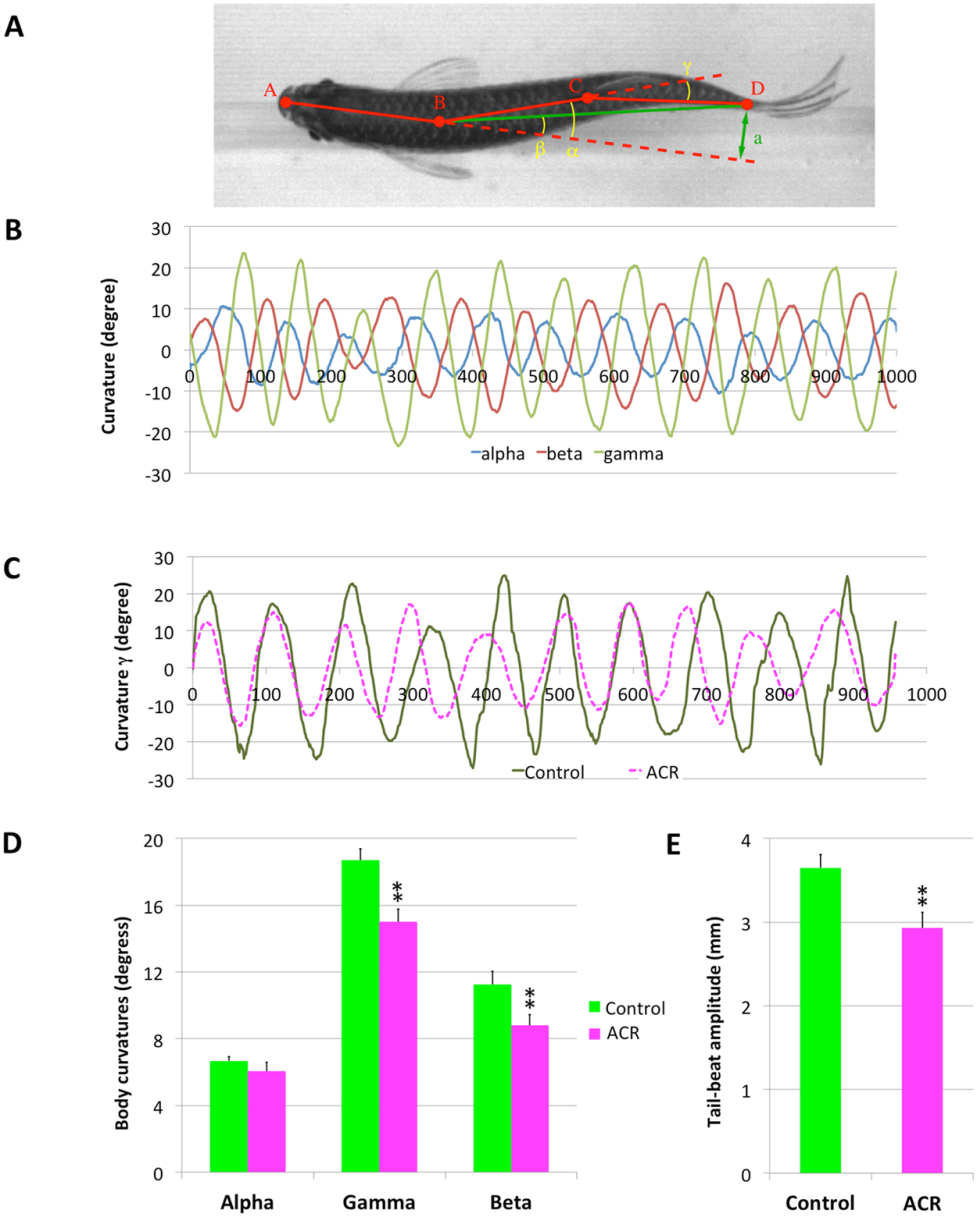
Kinematic analysis of the swimming showing ataxic gait in acrylamide (ACR)-exposed zebrafish. (**A**) For each frame, the kinematic analysis software divides the fish body in three segments of the same length, measuring the angles α, β and γ. (**B**) Time-course of angles α, β and γ from a representative control zebrafish (time in milliseconds). (**C**) Representative kinematic traces (angle γ) of control and ACR-treated fish. (**D**) Average curvature measured by angles α, β and γ over half a cycle of bending for control and ACR-exposed fish (mean ± SE; n = 7 for control and n = 6 for ACR-exposed fish). (**E**) Average tail-beat amplitude for control and ACR-exposed fish (mean ± SE; n = 7 for control and n = 6 for ACR-exposed fish). **p < 0.01 Student’s t-test.

The presence of gait abnormalities is one of the main neurological hallmark of ACR poisoning in humans and animal models^4,15–17^. Although some parameter related with the motor function, as total distance moved in the open field test, were initially assessed in the model for acute ACR neurotoxicity recently developed in adult zebrafish, the presence of gait abnormalities was not determined. In order to evaluate if the recently developed chemical model of ACR acute neurotoxicity exhibits this clinical sign, a kinematic analysis of the swimming of the fish needs to be performed. First of all, we developed *ZebraGait*, a kinematic analysis software for determining potential gait impairment in fish swimming inside a water tunnel at different speeds. Then, the steady swimming of control and ACR-exposed fish in a water tunnel at 2 body-lengths per second (BL/s) was recorded with a high-speed camera at 1000 frames per second (fps), and fragments of 1 s of the video were further analyzed with *ZebraGait*. For each frame, the software divided the body in three segments of the same length, corresponding approximately to the head, trunk and tail of the fish, measuring the angles between these segments (Fig. 1A,B; see M&M section for more details). The following kinematic parameters have been determined for characterizing the body curvature of the fish during the swimming: (1) curvatures of the three selected angles, (2) *tail-beat amplitude* and (3) the mean *tail-beat frequency* during all the cycles of the video fragment. Whereas no differences were found in the tail-beat frequencies of the movements of the different body segments between control (head-trunk: 10.13 ± 0.58 Hz; trunk-tail: 10.26 ± 0.58 Hz; head-tail: 10.18 ± 0.58 Hz) and ACR-exposed fish (head-trunk: 10.61 ± 0.58 Hz; trunk-tail: 10.67 ± 0.56 Hz; head-tail: 10.66 ± 0.54 Hz), the average angle γ over half a cycle of bending was significantly lower in ACR-exposed fish (Fig. 1C,D). Also, the average angle β (Fig. 1D) and the tail-beat amplitude (Fig. 1E) were significantly reduced in ACR-exposed fish. Whereas swimming of the animals in the water tunnel was also recorded at higher water speeds (3 and 4 BL/s), fish exhibited a clear difficulty to maintain the position in the center of the tunnel, making the analysis impossible. These results confirm the presence of mild-to-moderate gait abnormalities in the developed zebrafish model for ACR acute neurotoxicity, a result consistent with the altered gait reported in mammalian species^4,15,17^. *ZebraGait* should be also a valuable tool for assessing changes in the kinematic of the gait in zebrafish models other pathologies exhibiting abnormal gait, including multiple sclerosis, Parkinson’s disease or myasthenia gravis.

### ACR-exposed zebrafish exhibits negative scototaxis

**Figure 2:**
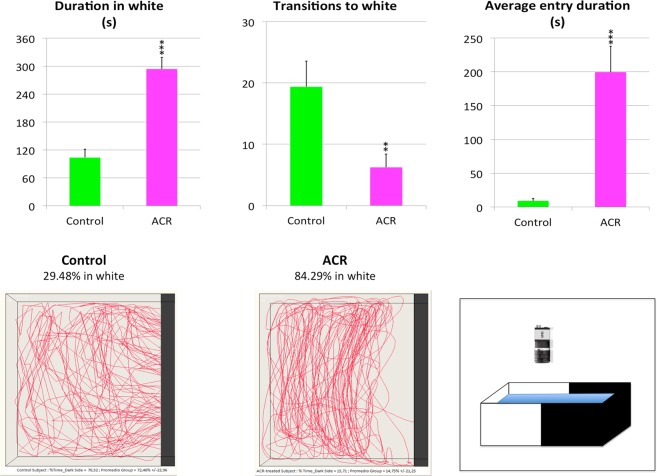
Behavioral effects of 3 days exposure to 0.75 mM acrylamide (ACR) on zebrafish tested in the dark-light paradigm (DLT). Behavioral parameters assessed in standard 6-min DLT, as well as a cartoon of the experimental tank divided into two equal virtual zones, white and black, and representative traces of control and ACR-treated fish. Mean and standard error from two independent experiments (n = 17 for control and n = 18 for ACR-exposed fish). ***p < 0.001, Student’s t-test.

Behavioral phenotype of the acute ACR zebrafish model was characterized in a previous study by using the novel tank test (NTT) and open field test (OFT) paradigms^14^. Results obtained in that study strongly suggested an anxiety comorbid with depression phenotype. Results of the dark/light test (DLT), an experimental paradigm designed to assess scototaxis, for control and ACR-treated fish are shown in Fig. 2. First of all, a significant decrease in the swimming speed was found in ACR-exposed fish (8.54 ± 0.95 cm/s; p < 0.05) compared to the controls (14.78 ± 2.28 cm/s), a result consistent with the reported hypolocomotion in the NTT and OFT^14^. ACR induced negative scototaxis, spending more time in the white zone (p < 0.001). Although the number of transitions of ACR-exposed fish to the white area was lower than the control values (p < 0.01), the duration of each entry was significantly higher in ACR-treated animals (p < 0.001). Representative traces generated by Ethovision XT 13.0 software clearly support the dramatic effect of ACR on the white zone preference (Fig. 2 and Supplementary Video S1).

The negative scototaxis found in ACR-exposed fish supports the development of an anxiety comorbid with depression phenotype in animals acutely exposed to ACR. Thus, the neurotransmitter profile and the behavioral phenotype found in the acute ACR neurotoxicity model are similar to the reported for *Vmat2* zebrafish mutants, with a significant depletion of the monoaminergic neurotransmitters, positive geotaxis and negative scototaxis^18^. Moreover, and similarly to the effect of ACR, zebrafish exhibiting serotonin depletion after treatment with the TPH inhibitor PCPA also exhibited positive geotaxis and negative scototaxis^19^.

### GSH depletion, but not oxidative stress, in the brain of ACR-exposed zebrafish

Oxidative stress in the brain has been linked to the neuronal cell death associated with neurodegeneration^20^. One of the primary events in ACR-induced neuropathy is a significant decrease in the intracellular GSH pool in the brain^7,10^, and this effect can finally result in the generation of oxidative stress and neurodegeneration after subchronical exposures^21–23^. In fact, oxidative stress has been proposed as the main mechanism leading to ACR neurotoxicity^10,23,24^, and many different antioxidant compounds have been suggested as potential antidotes against this syndrome^21,25,26^. In order to determine the presence of oxidative stress in the brain of the ACR-treated zebrafish, the decrease of the reduced glutathione (GSH) intracellular pool and the presence of ROS-mediated lipid peroxidation in the brain were evaluated. In spite that a significant decrease in GSH levels was found in the brain of the treated fish (2.656 ± 0.158 vs 1.162 ± 0.098 nmol GSH/mg ww for control (n = 9) and ACR-exposed (n = 8) fish, respectively; p = 1.18 × 10^−6^, Student’s t-test), no differences in lipid peroxidation levels were found between control and ACR-treated fish (41.68 ± 2.85 vs 51.69 ± 8.05 pmol MDA/mg ww for control (n = 7) and ACR-exposed (n = 7) fish, respectively; p = 0.264, Student’s t-test). Similarly to what has been reported in other species, ACR induces a significant decrease in GSH levels in the brain. Generation of oxidative stress involves a sequence of molecular events, where cytosolic GSH depletion should be followed by the depletion of mitochondrial GSH and then, by an increase in ROS production by the mitochondria. There is a delay of about 48 h between the full depletion of cytosolic GSH and the depletion of the mitochondrial stores^27^. Thus, whereas 72 h of exposure to ACR is time enough for depleting GSH levels, this time might be too short to significantly increase the ROS levels.

The fact that the developed model exhibits gait abnormalities and behavioral effects, but not oxidative stress, demonstrates that generation of oxidative stress is not a relevant key event in the Adverse Outcome Pathway (AOP) resulting in ACR neurotoxicity.

### Neurobehavioral phenotype of ACR-exposed zebrafish is reversible

**Figure 3:**
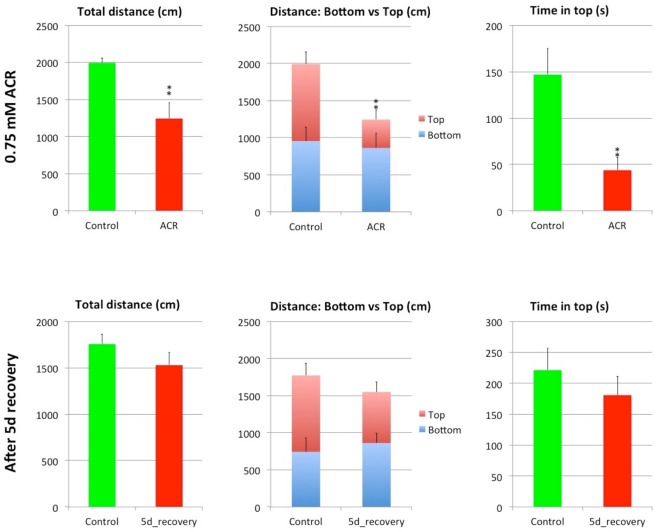
Hypolocomotion and positive geotaxis induced by 3 d exposure to 0.75 mM ACR are completely abolished after 5 days recovery period in clean fish water. Results of total distance, distance moved by zone (bottom and top of the tank), and time spent in the top of the tank the bottom in the Novel Tank Test (NTT). Mean and standard error from two independent experiments (n = 12). **p < 0.01, Student’s t-test.

Neurodegeneration is an irreversible phenotype^12^. In order to determine if the phenotype of the adult zebrafish model of ACR acute neurotoxicity was consistent with a neurodegenerative process, the reversibility of the neurobehavioral phenotype was evaluated. A set of recovery experiments were performed by transferring the ACR-exposed fish, at the end of the exposure period, to clean fish-water. No lethality was found after 10 days in clean fish-water. Moreover, a full recovery of the hypolocomotion and the positive geotaxis, the main behavioral parameters altered in ACR-exposed fish in the novel tank test (NTT) paradigm, was observed 5 days after transferring the fish to clean fish water (Fig. 3). These results support the absence of neurodegeneration, indicating also that the grade of severity of the neurotoxic syndrome in zebrafish was from mild to moderate.

All the results presented in this manuscript strongly support the construct validity of the acrylamide acute neurotoxicity model developed in zebrafish. Moreover, the fact that gait abnormalities and other neurobehavioral effects are present in the absence of oxidative stress strongly support the hypothesis of the impairment of the presynaptic function as the main key event in the development of ACR neurotoxicity. Finally, the software developed for assessing the kinematics of the fish swimming should be an extremely useful tool for neuropathology research using zebrafish as animal model.

## Material and Methods

### Zebrafish experiments

Wild-type zebrafish were obtained from Piscicultura Superior (Barcelona, Spain) and maintained in fish water [reverse-osmosis purified water containing 90 mg/L Instant Ocean® (Aquarium Systems, Sarrebourg, France), 0.58 mM CaSO_4_ · 2H_2_O] at 28 ± 1 °C under a 12 L:12D photoperiod.

For developing the model of ACR acute neurotoxicity, adult zebrafish (≈50:50 male:female ratio; standard length: 3.8–4.2 cm) were exposed for 72 h to 0.75 mM ACR (Sigma-Aldrich, St. Louis, MO) in fish water. Control fish were maintained in fish water under identical conditions. Experimental solutions were renewed after 48 h of exposure.

For sample collection fish were euthanized by inducing hypothermic shock in ice-chilled water (2° to 4 °C). Brain tissue was immediately excised and pooled (4 brains/sample) and stored at −80 °C for further analyses.

All procedures were approved by the Institutional Animal Care and Use Committees at the CID-CSIC and conducted in accordance with the institutional guidelines under a license from the local government (agreement number 9027).

### Video recording setup

Experiments for assessing the swimming kinematics were performed in a 170 mL miniature swim tunnel (Loligo® Systems, Denmark; Supplementary Fig. S1). Standard length of the fish was measured three days before the swimming experiments, in order to avoid this source of stress during the kinematic analysis. The day of the experiment, the swim tunnel was submerged in a 20 L water tank supplied with fish water. Water flow velocities were calibrated using a Flow Tracking system (DPTV), based on 2D video tracking of green laser illuminated flourescent spheres with neutral buyancy (Loligo® Systems, Denmark). One fish was placed in the swim tunnel between two honeycombs and acclimated at a water velocity of 1 body length (BL)/s for 30 min. After acclimation, the fish was forced to swim against the current of 2 BL/s, a mild to moderate swimming speed in zebrafish. The videos of the fishes were recorded with a high-speed Photron Fastcam Mini UX100 (Photron USA Inc., San Diego, CA, USA; Supplementary Fig. S1) at 1280 × 312 pixel resolution using a Sigma 50 mm F1.4 DG lens at 1000 frames per second (Supplementary Video S2 Video). The tunnel was indirectly illuminated by a white LED (Multiled LT-V9, GS Vitec, Germany). The light intensity in the tunnel was measured with a Iso-Tech-1332A digital illuminance meter (Iso-Tech LTD, England) and adjusted to 486 lux. After each experiment, the videos were retrieved from the memory of the camera and saved as an AVI file without compression.

### Kinematic analysis software

The video analysis tools have been developed in Python using the OpenCV libraries to process the images. In a pre-process, the images are normalized, their contrast is enhanced to eliminate irrelevant parts of the fish such as the fins or the tail, and a Gaussian filter is applied to reduce the noise. Then, the user marks the area of interest in the image (i.e., the fish chamber in the flow tunnel). To speed up the image processing, the selected area is further clipped using motion detection techniques^28^. In this way, the computation is focused on the relevant parts of the captured image. The process continues with a binarization of the area of interest using the Otsu algorithm^29^, which automatically calculates the optimal threshold to separate objects and background. The blobs in the resulting binary images are detected and the one fulfilling a particular set of parameters with respect to its size and shape is assumed to be the fish. The skeleton of this blob is computed using the Zhang-Suen thinning algorithm^30^ to identify the dorsal of the fish. Several checks are performed in the different steps of the process to ensure that the final result is valid. These checks consider aspects such as the number of detected blobs, their sizes, or the inclusion of the skeleton in a particular blob. If any of the checks fails, the frame is considered invalid. Only videos with less than 10% of invalid frames are used in the evaluation.

Once the dorsal of the fish is identified, we extract numerical values to evaluate the fish behavior. In our process, the dorsal is approximated with three straight segments, connecting four points equidistributed along the dorsal (points A, B, C, and D in Fig. 1A). These segments provide a good approximation of the posture of the fish. From these values, we calculated three main parameters (see Fig. 1A): (1) *curvatures*: measured with the head-trunk angle, β, the head-tail angle α, and the trunk-tail angle, γ, (2) the *tail-beat amplitude*, *a*, computed as the distance between the end of the caudal peduncle and the line including the head segment $\overline{{\rm{AB}}}$, and (3) the *mean tail-beat frequency*, using data from all the cycles of the video fragment. Supplementary Video S3 summarizes all the different steps of the analysis performed with the software.

### Behavioral analysis

All testing was performed in an isolated behavioral room at 27–28 °C. Animals (≈50:50 male:female ratio) were brought to the behavioral room 24 hours before beginning the test, to acclimate to the environment, and then, behavioral testing was conducted between 10:00 and 15:00 h. The dark-light test (DLT), used to assess anxiety and/or depression, was performed using an experimental setup allowing monitoring and recording 2 fish simultaneously. The DLT was performed in two experimental tanks (38 cm length, 24 cm width, 28 cm height) with one horizontal half made of white acrylic and the other half made of black acrylic. Each experimental tank contained 9 L (10 cm height) of fish water at 28 °C. Two anti-flicker LED tubes (TUT8-ST28-NFL; AS de LED®, Valencia, Spain) mounted on both sides of the test tanks provided uniform illumination for video-recording. Light intensity in the dark and light zones, measured with a Iso-Tech-1332A digital illuminance meter (Iso-Tech LTD, England), was in the range of 250–300 lux. The first 6 min of the trial were video-recorded (AVI format, 30 fps) with the uEye Cockpit software (version 4.90; IDS GmbH, Germany) controlling the GigE cameras (UI-5240CP-NIR-GL, IDS GmbH, Germany) placed on top of the testing tanks. The recorded videos were analyzed by Ethovision XT 13.0, and the time spent in the white zone (s), number of transitions to the white zone and the average duration of the entries to the white zone duration in the white mobility state (s) were determined.

The novel tank test was performed according to Faria *et al*.^14^ After acquisition, videos were analyzed by Ethovision XT 13.0 (Noldus, Wageningen, the Netherlands), and the total distance travelled (cm), distance travelled in the top and in the bottom (cm) and the time spent in the top (s) were determined.

### GSH determination

Zebrafish brain tissue was homogenized in ice cold 0.1 M phosphate buffer pH 7.4 at a 12 mg/mL (tissue weight/buffer volume) rate, and then centrifuged at 10,000 × g, 4 °C for 10 minutes. The supernatant was collected for reduced glutathione (GSH) quantification. Levels of GSH were determined according to Faria *et al*.^31^. In brief, 0.1 mM of monochlorobimane (mCB) and 1 U/mL of Glutathione S transferese (GST) was added to each sample and left to incubate at 25 °C, protected from light for 150 minutes. The resulting GSH-mCB complex was measured fluorometrically at an excitation/emission wave length of 360 nm/460 nm using a microplate reader (Synergy 2 multi mode, ®BioTek Instruments Inc., Winooski, VT, USA). The content of GSH was then extrapolated from a GSH standard curve determined under the same conditions as the samples, and the final results were normalized by total tissue weight (mg) and expressed as nmol/mg of tissue wet weight (ww).

### Lipid peroxidation

Zebrafish brains were pooled (n = 2), weighed and homogenized in ice cold 0.1 M phosphate buffer with 150 mM KCl and 0.1 mM ethylenediamine-tetraacetic acid, disodium, salt, dihydrate (EDTA) to a final 30 mg/ml tissue weight/buffer volume relation and immediately analysed. Lipid peroxidation (LPO) was determined by quantifying the levels of malondialdehyde (MDA), according to Esterbauer and Cheeseman^32^. Brain homogenates were incubated with 5 mM 1-methyl-2-phenylindole in acetonitrile:methanol (3:1 v/v), 5.55% of HCl and 0.01% BHT at 45 °C, for 40 minutes. Absorbance was read at 560 nm and MDA content in each sample was extrapolated from the standard curve of 1,1,3,3-tetramethoxypropane (TMP) treated under similar conditions as samples. The final results were normalized by mg ww of each sample and expressed as pmol/mg.

## Supplementary information

Supplementary Information

Supplementary Video S1

Supplementary Video S2

Supplementary Video S3

## Data Availability

The source code of the ZebraGait software is released under GNU v3.0 license, and can be accessed at https://github.com/jmporta/ZebraGait.
