# Supplementary material for: Further characterization of the zebrafish model of acrylamide acute neurotoxicity: gait abnormalities and oxidative stress
Source: Sci Rep. 2019 May 8;9:7075. doi: 10.1038/s41598-019-43647-z (PMC6506514; doi:10.1038/s41598-019-43647-z)
Supplement: Supplementary file 1 — Supplementary Information [file 41598_2019_43647_MOESM1_ESM.pdf]

Supplementary Information

Further characterization of the zebrafish model of acrylamide acute neurotoxicity: gait abnormalities and oxidative stress

Melissa Faria, Arnau Valls, Eva Prats, Juliette Bedrossiantz, Manuel Orozco, Josep M. Porta, Leobardo Manuel Gómez-Oliván, Demetrio Raldúa*,

*Address correspondence to: Demetrio Raldúa, Institute of Environmental Assessment and Water Research (IDAEA-CSIC), Jordi Girona 18, 08034 Barcelona, Spain. Telephone: +34-93-4006138. E-mail: drpqam@cid.csic.es

Title of file for HTML: Supplementary Information
Description: Supplementary Figures,

Title of file for HTML: Supplementary Video SV1
Description: **Representative behavior of the control and ACR-treated zebrafish in the dark-light test**.

Title of file for HTML: Supplementary Video SV2
Description: **Adult zebrafish swimming at 2 BL/s in a water tunnel. The fish was recorded during 1 s at 1000 fps with a high-speed Photron Fastcam Mini UX100 for further kinematic analysis with *ZebraGate*.**

Title of file for HTML: Supplementary Video SV3
Description: **Different steps of the kinematic analysis performed by *ZebraGait* of a video sequence (1 s, 1000 fps) of a representative fish swimming at 2 BL/S in a water tunnel.**

.

Supplementary Information

Supplementary Figures

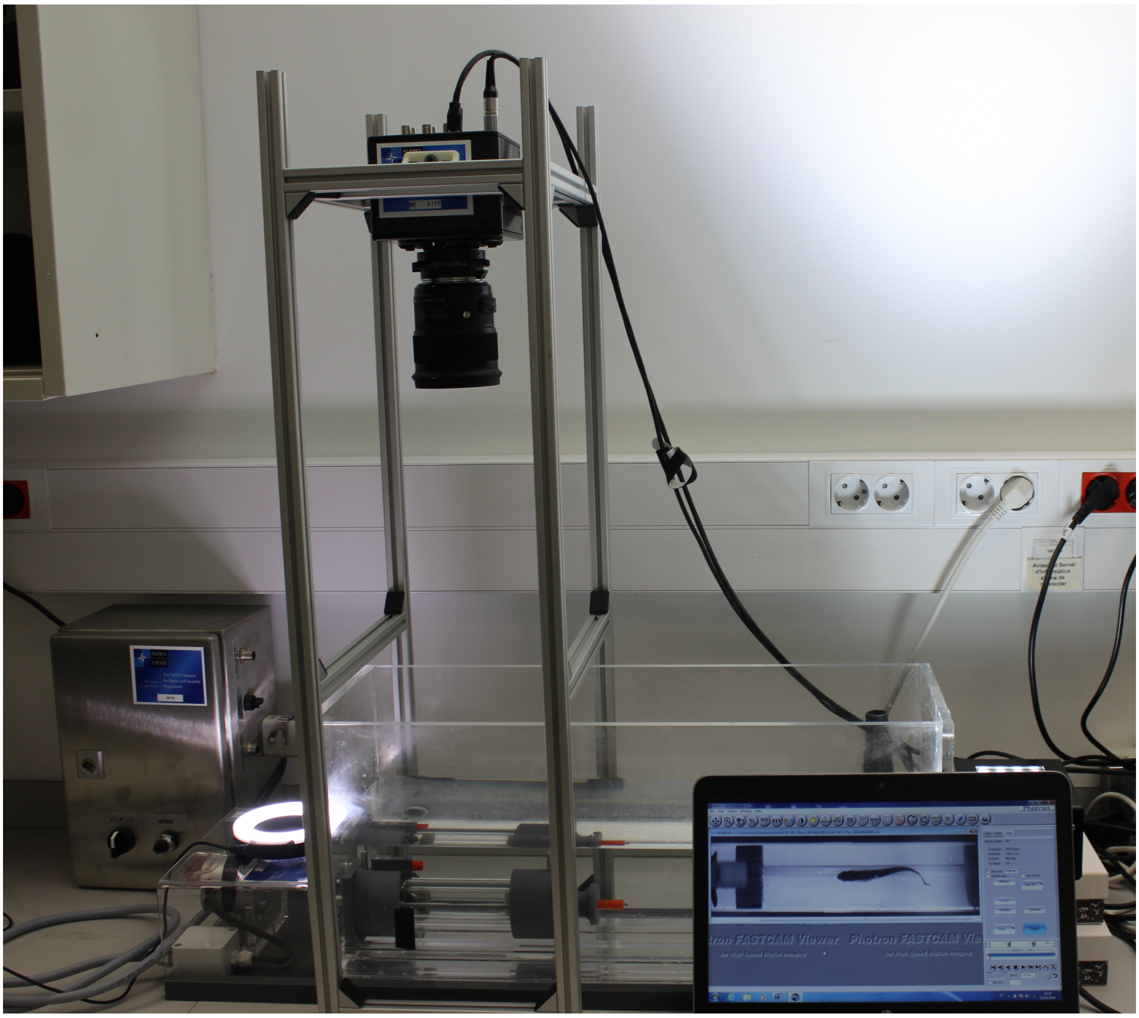

Supplementary Figure S1. Experimental setup used for for video-recording the fish swimming in a water tunnel at 1000 frames per second.
